# Supplementary material for: Aging and Alzheimer’s disease have dissociable effects on local and regional medial temporal lobe connectivity
Source: Brain Commun. 2023 Sep 14;5(5):fcad245. doi: 10.1093/braincomms/fcad245 (PMC10521906; doi:10.1093/braincomms/fcad245)
Supplement: fcad245_Supplementary_Data [file fcad245_supplementary_data.pdf]

SUPPLEMENTARY METHODS

Anatomical Preprocessing in *fMRIPrep*

First, each participant's T1-weighted (T1w) image was corrected for intensity non-uniformity using the N4 algorithm (Tustison et al., 2010). Each T1w image was then skull-stripped with a Nipype (Gorgolewski et al., 2011) implementation of the ANTs brain extraction workflow, using OASIS30ANTs as the target template. Brain-extracted T1w scans were subsequently registered to the brain-extracted 1-mm MNI152NLin6Asym template using SyN diffeomorphic registration (ANTs 2.2.0; Avants & Gee, 2004; Avants et al., 2008) and brain surfaces were reconstructed using FreeSurfer 6.0.1 (Dale et al., 1999; http://surfer.nmr.mgh.harvard.edu). Subject-level CSF, WM, and gray matter (GM) segmentations were performed on brain-extracted T1-weighted scans using FSL *fast* (FSL 5.0.9; Zhang et al., 2001).

Amyloid PET Analysis

Amyloid PET data were processed with in-house software. First, attenuation-corrected dynamic image frames were motion-corrected using *mcflirt* rigid-body registration (FSL 5.0.9; Jenkinson et al., 2002; Smith et al., 2004). The resulting motion-corrected PET frames were averaged and aligned with participants' T1-weighted structural MRI scans using ANTs rigid-body registration with a mutual information metric (Avants & Gee, 2004; Avants et al., 2008). Each anatomical MRI was segmented into cortical, subcortical, and cerebellar ROIs using a multi-atlas segmentation method (Asman & Landman, 2013; Wang et al., 2013). Mean tracer uptake in the cerebellar gray and white matter was computed and used as a reference to generate a standardized uptake value ratio (SUVR) map for the entire brain. A composite ROI consisting of the middle frontal, anterior cingulate, posterior cingulate, inferior parietal, precuneus, supramarginal, middle temporal, and superior temporal cortical regions was used to compute a global SUVR for amyloid scans (Landau et al., 2013).

ICA-Based Denoising Protocol

A single rater (SH) reviewed all ICA-AROMA classifications and corrected classification inaccuracies. Spatial maps, time courses, and power spectra of every component from every subject were inspected. Scanner noise components were identified by two criteria: (1) majority of spatial activation outside the gray matter, and (2) distinct power spectrum pattern, dominated by high-frequency spikes – generally above 0.11 Hz – with little to no power represented by lower frequencies (i.e., < 0.10 Hz). Cardiovascular and respiratory noise sources were identified based on the guidelines detailed in Griffanti et al. (2017). Head-movement-related components were largely based on the ICA-AROMA classifications (Pruim et al., 2015) and were generally characterized by slow drifts or sharp signal spikes. Only unambiguous noise components were marked for removal. SH has extensive experience with manual ICA-based denoising and attains approximately 90% intra-rater classification consistency after a 2-week delay (Hrybouski et al., 2021). The dominant head motion artifacts (e.g., global signal drifts with spatial maps localized exclusively to the skull) were removed using the 'aggressive' denoising option in *fsl_regfilt*, while all other artifacts were removed using the 'soft' denoising option in *fsl_regfilt* (Beckmann & Smith, 2004; Griffanti et al., 2014).

ROI-based Representation of the Extended MTL Network

Extra-MTL ROIs with positive functional connectivity (FC) to the MTL were selected from the 400-region 17-Network Schaefer et al. (2018) parcellation. Cortical regions with connectivity to the MTL were identified in normal agers only [i.e., CU young, middle-aged, and Aβ− older participants]. To test for the presence of FC to the MTL, we performed 4 sets (one per each MTL ROI: left anterior, right anterior, left posterior, right posterior) of one-sample positive-sided *t*-tests [FDR-corrected, $q < 0.05$] on Fisher-transformed subject-level Pearson correlation coefficients, representing that segment's FC to each of the 393 non-MTL Schaeffer ROIs. Seven Schaeffer ROIs were excluded because of substantial (>15%) spatial overlap with our anterior or posterior tau-based MTL seeds. To ensure that we did not miss any major cortical regions with

FC to the MTL, we also performed seed-to-voxel network identification. Here, one-sample permutation tests (5,000 permutations) for positive connectivity to the bilateral anterior and posterior tau-based MTL ROIs were performed on Fisher-transformed subject-level voxelwise seed-to-voxel connectivity maps (Conn 20.b; Whitfield-Gabrieli & Nieto-Castanon, 2012). The Threshold-Free Cluster Enhancement (TFCE) method with the FDR ($q < .05$) correction for multiple hypothesis testing was used in these voxelwise tests (Benjamini & Hochberg, 1995; Smith & Nichols, 2009). We considered a given Schaeffer ROI as a part of the broader MTL-associated functional system if it was functionally connected to at least one of the MTL ROIs in the ROI-to-ROI network identification or if more than 40% of that ROI's voxels corresponded to a statistically significant cluster in the voxelwise network identification method. In total, we identified 221 Schaeffer ROIs with positive functional connectivity to the MTL. Together with 4 seed regions, these 221 Schaeffer ROIs (225 ROIs in total) were used in all subsequent analyses of the MTL network function (Fig. 2a in the main text).

Estimation of Network Architecture Using Graphical SCAD

First, bivariate correlation matrices were computed for all participants in a group. For *normal agers*, those correlation matrices were then Fisher-transformed and averaged such that each decade of human lifespan had equal weight on the final connectivity structure. For other groups, simple averaging across all participants was performed after applying Fisher transformations. The resulting average Z-connectivity matrices were converted into the correlational connectivity matrix and used as covariance sources in SCAD-based network estimation. Graphical SCAD optimization relies on two tuning parameters: α and ρ. To minimize the Bayes risk, Fan and Li (2001) recommend $\alpha = 3.7$. The second tuning parameter, ρ, was selected from a set of $\rho = \{e^{-8.0}, e^{-7.8}, e^{-7.6}, \dots, e^{0}\}$ by minimizing the Bayesian Information Criterion (Fan et al., 2009; Zhu & Cribben, 2018). Custom MATLAB scripts employing the QUIC optimizer were used to solve the graphical SCAD problem (Hsieh et al., 2014).

SUPPLEMENTARY FIGURES

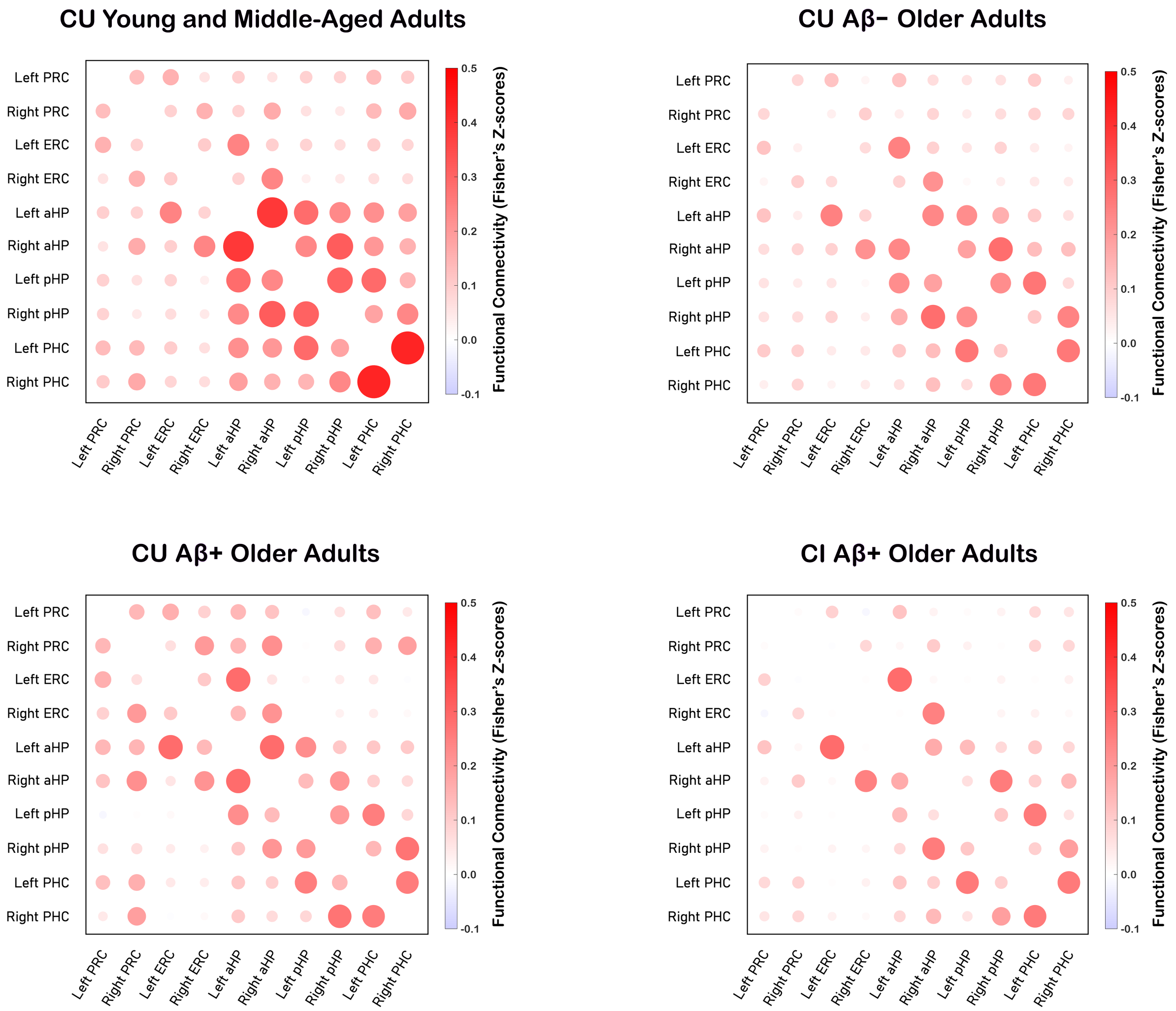

Supplementary Figure 1. Mean intra-MTL connectivity, separated by group. In young and middle-aged adults ($N_{young/middle}$ = 36), functional connectivity scores were corrected for sex differences. In older adults ($N_{A\beta- CU}$ = 80, $N_{A\beta+ CU}$ = 23, $N_{A\beta+ CI}$ = 40), functional connectivity scores were corrected for differences in age, sex, and head movement. Young/middle-aged functional connectivity scores represent intra-MTL connectivity at 39 years of age; functional connectivity scores for the three older groups represent intra-MTL connectivity at 72 years of age. Abbreviations: PRC = perirhinal cortex; ERC = entorhinal cortex; PHC = parahippocampal cortex; aHP = anterior hippocampus; pHP = posterior hippocampus; MTL = medial temporal lobe; Aβ− = amyloid-negative; Aβ+ = amyloid-positive; CU = cognitively unimpaired; CI = cognitively impaired.

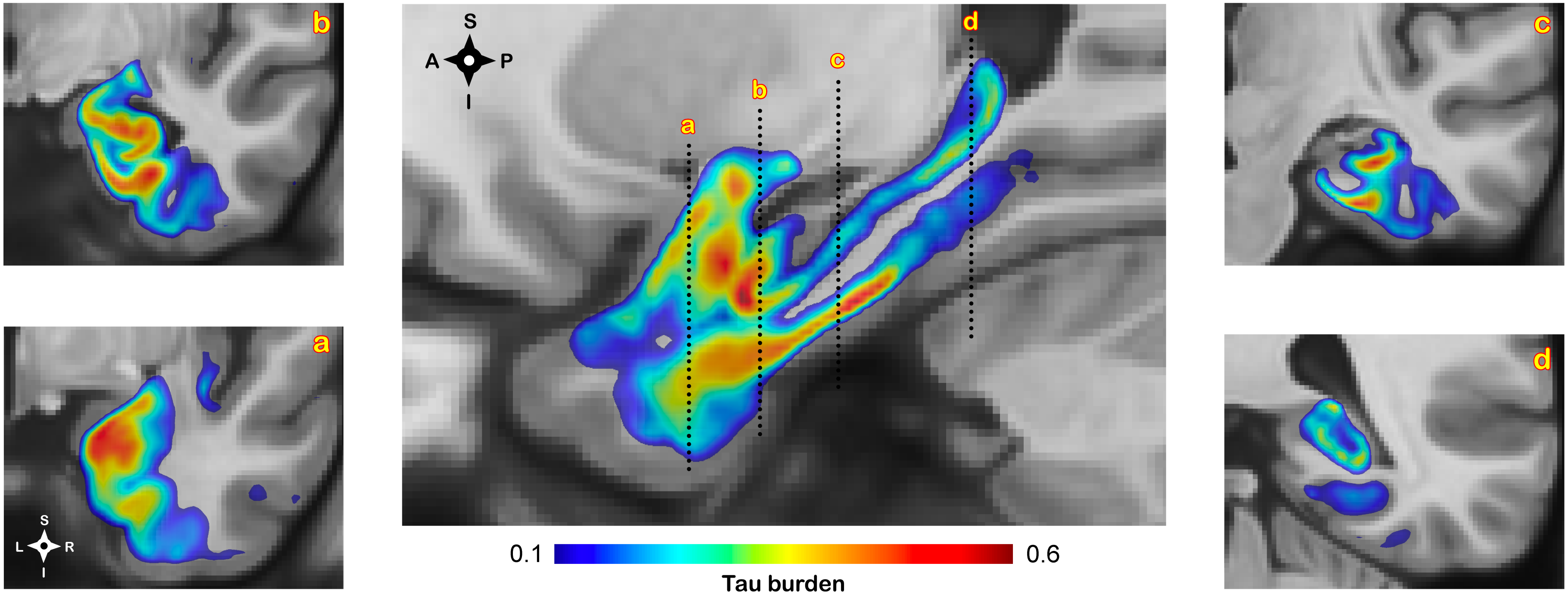

Supplementary Figure 2. Coronal and sagittal planes depict the overall Medial Temporal Lobe (MTL) neurofibrillary tangle burden, derived from a serial histological examination of 15 MTL specimens (for detailed methodology see, Yushkevich et al., 2021). These tau maps were used to create tau-based ROIs for MTL-AT and MTL-PM connectivity analyses.

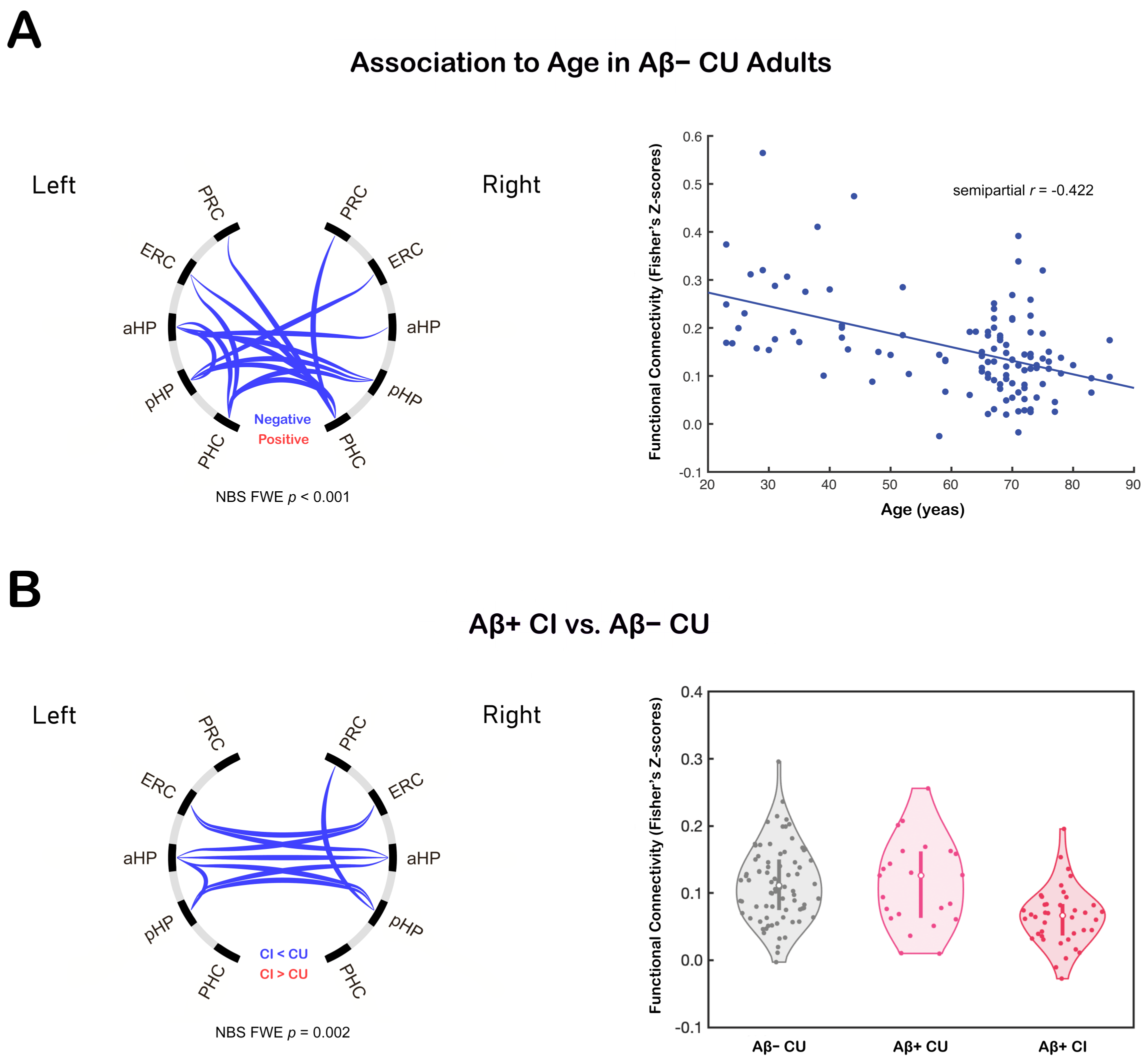

Supplementary Figure 3. The effects of age and AD on intra-MTL functional connectivity. **(A).** The connectogram on the left illustrates statistically significant correlations between age and intra-MTL connectivity in normal agers (*N* = 116). The scatter plot on the right depicts this cluster's mean connectivity scores as a function of age. **(B)** The connectogram on the left represents functional connectivity differences between Aβ-negative cognitively normal older adults ($N_{A\beta- CU}$ = 80) and age-matched Aβ-positive cognitively impaired patients with early Alzheimer's disease ($N_{A\beta+ CI}$ = 40). The violin plot on the right depicts this cluster's mean functional connectivity scores in controls, individuals with preclinical AD, and patients with symptomatic AD. Statistical significance was assessed using GLMs with cluster-like NBS-based correction for multiple comparisons. Abbreviations: PRC = perirhinal cortex; ERC = entorhinal cortex; PHC = parahippocampal cortex; aHP = anterior hippocampus; pHP = posterior hippocampus; MTL = medial temporal lobe; Aβ− = amyloid-negative; Aβ+ = amyloid-positive; CU = cognitively unimpaired; CI = cognitively impaired; GLM = general linear model; NBS = network-based statistic(s).

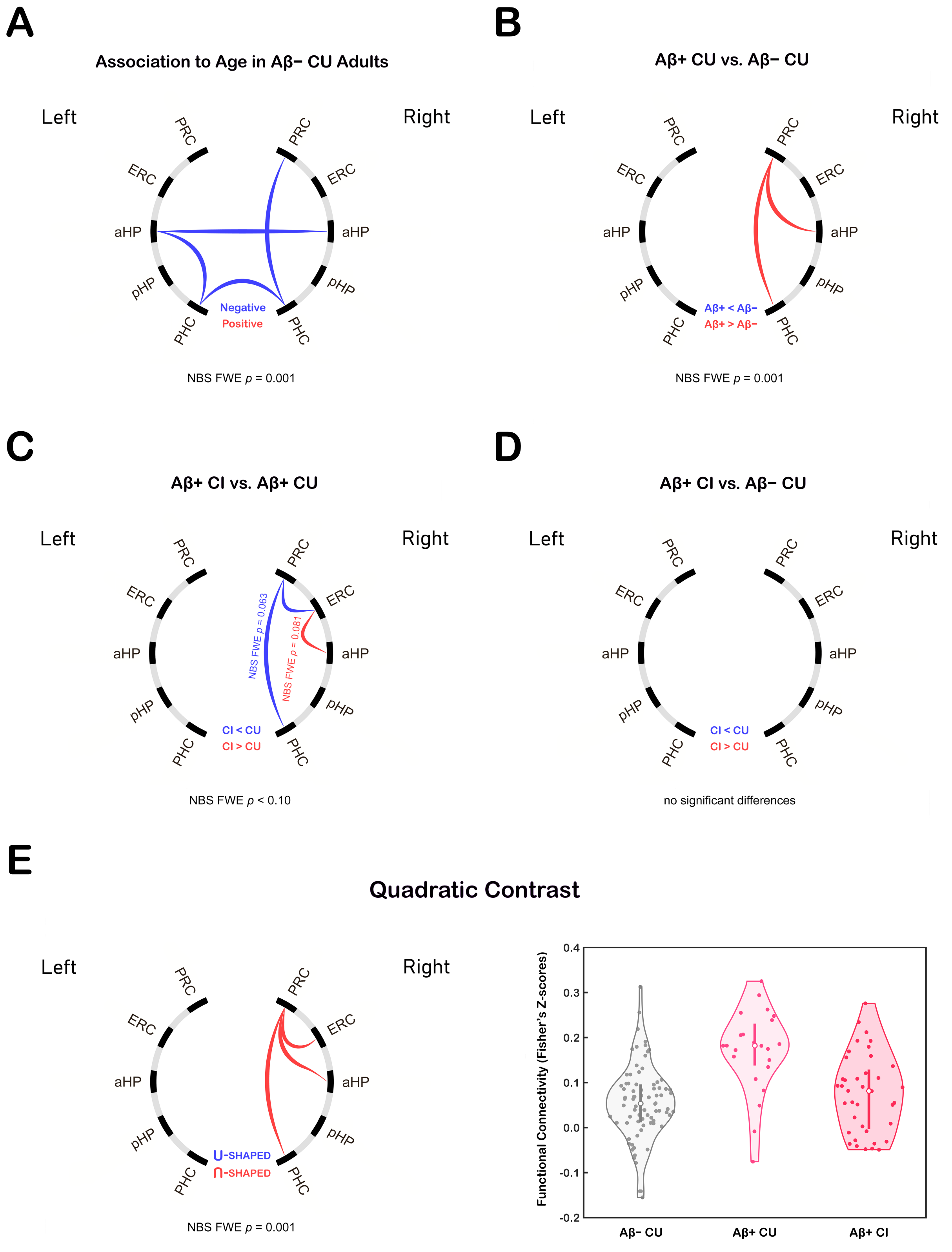

Supplementary Figure 4. This figure is a companion to Fig. 3 from the main text. Instead of canonical Pearson correlations, intra-MTL functional interactions were quantified using partial correlation coefficients, controlling for time courses from all other ASHS-T1 ROIs. Only those connections that represented *direct* intra-MTL functional interactions (see Fig. 1B in the main text) were analyzed. The NBS-based GLM approach was used to assess the statistical significance of each contrast/comparison ($N_{Aβ- CU}$ = 80, $N_{Aβ+ CU}$ = 23, $N_{Aβ+ CI}$ = 40). Connectograms depict the effects of **(A)** age and **(B-D)** AD progression on *direct* intra-MTL functional connectivity. **(E)** Left panel: cluster of intra-MTL connections with an inverted 'U-shaped' functional connectivity pattern in AD (GLM-based quadratic trend analysis; *N* = 143). Right panel: violin plot depicting this cluster's mean functional connectivity scores in controls, individuals with preclinical AD, and patients with symptomatic AD. Data points represent connectivity scores from individual participants. Abbreviations: PRC = perirhinal cortex; ERC = entorhinal cortex; PHC = parahippocampal cortex; aHP = anterior hippocampus; pHP = posterior hippocampus; Aβ− = amyloid-negative; Aβ+ = amyloid-positive; CU = cognitively unimpaired; CI = cognitively impaired; GLM = general linear model; NBS = network-based statistic(s).

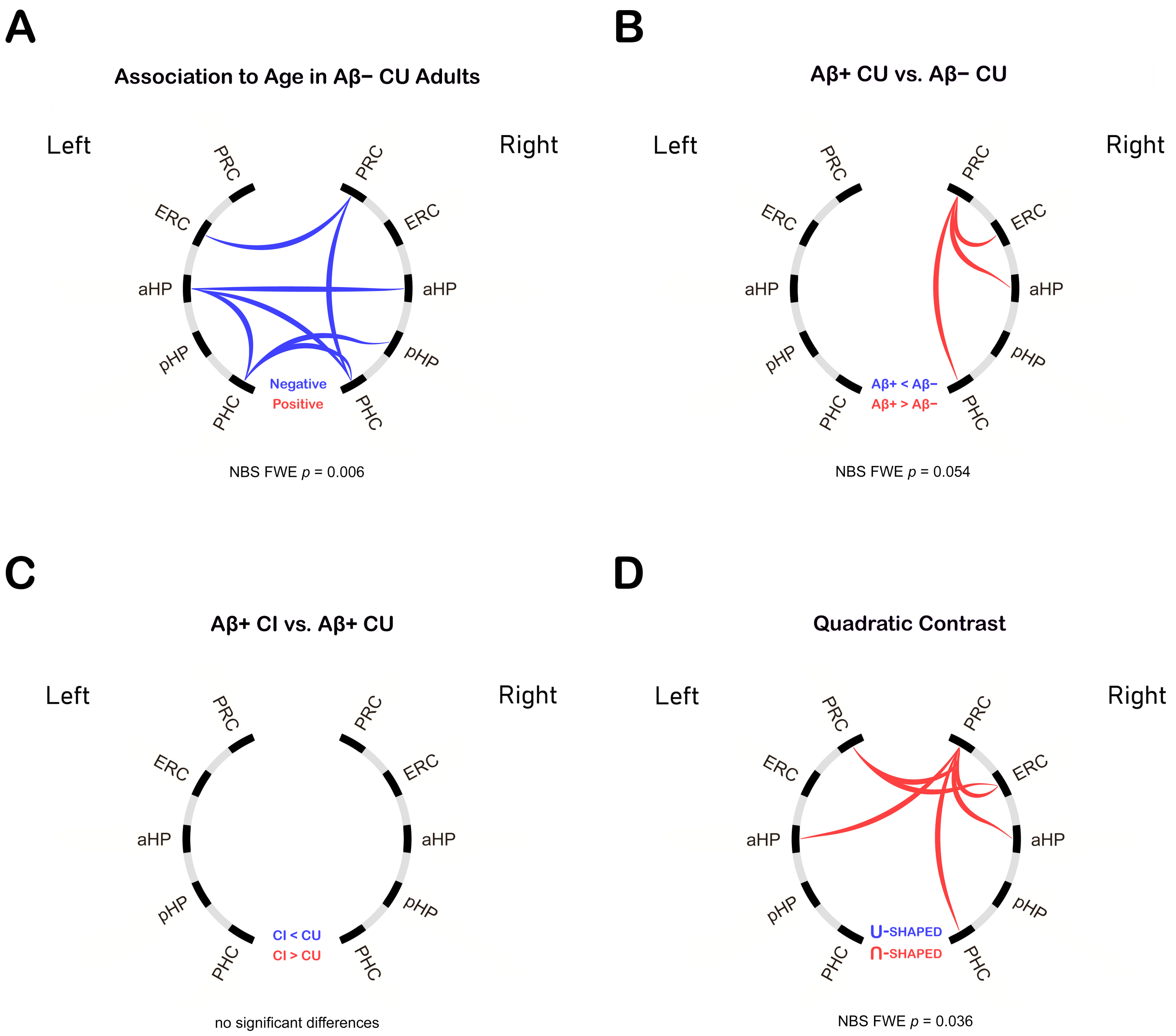

Supplementary Figure 5. The effects of **(A)** age and **(B-D)** AD progression on correlational measures of intra-MTL connectivity, after covarying for bilateral PRC thickness, ERC thickness, PHC thickness, and ICV-corrected aHP and pHP volumes ($N_{\text{panel } \mathbf{A}}$ = 116, $N_{\text{panel } \mathbf{B}}$ = 103, $N_{\text{panel } \mathbf{C}}$ = 63, $N_{\text{panel } \mathbf{D}}$ = 143). Statistical significance was assessed using GLMs with cluster-like NBS-based correction for multiple comparisons. Abbreviations: PRC = perirhinal cortex; ERC = entorhinal cortex; PHC = parahippocampal cortex; aHP = anterior hippocampus; pHP = posterior hippocampus; Aβ− = amyloid-negative; Aβ+ = amyloid-positive; CU = cognitively unimpaired; CI = cognitively impaired; GLM = general linear model; NBS = network-based statistic(s).

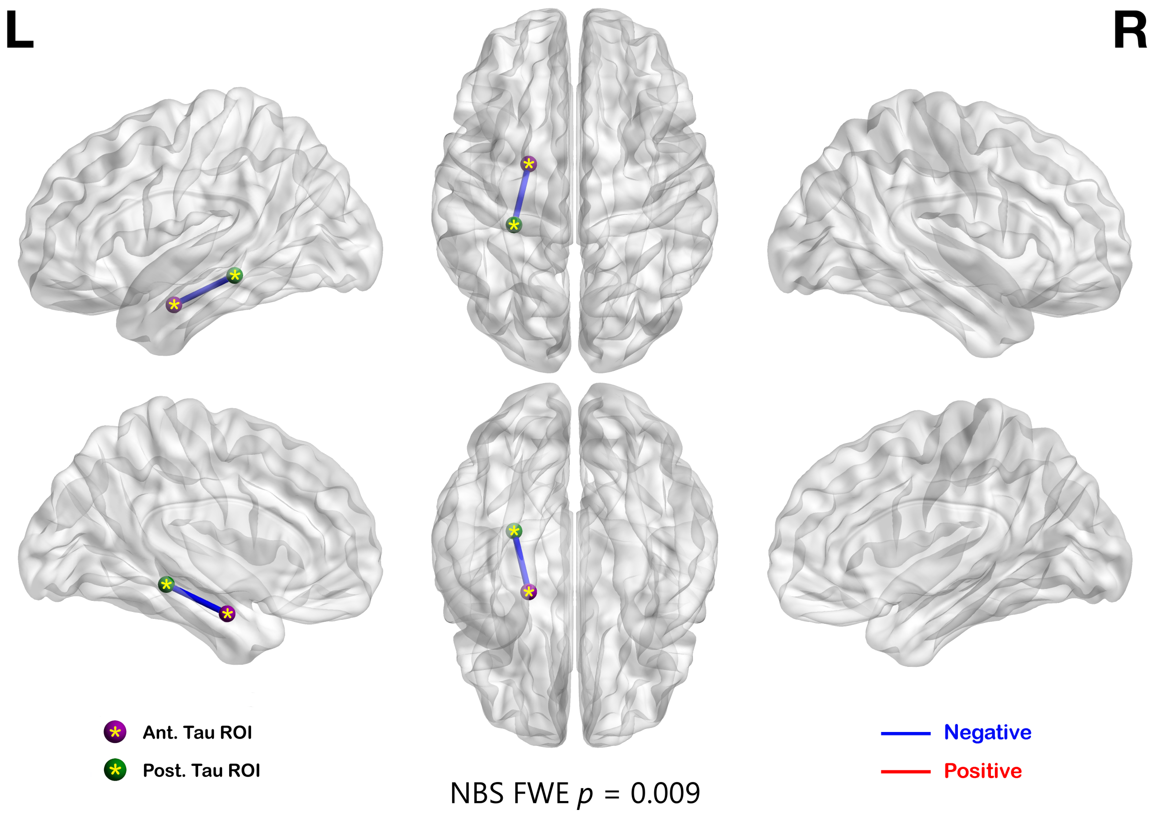

Supplementary Figure 6. The effect of age on AT-PM inter-network connectivity. Connection-level general linear models (GLMs) were used to test for the presence of linear relationships between connectivity strength and age in *normal agers* (N = 116). Cluster-like network-based statistical (NBS) framework was used to account for multiple hypothesis testing. A single connection, linking the left anterior and left posterior tau-based MTL ROIs with each other, displayed age-associated functional connectivity decline in amyloid-negative cognitively unimpaired adults.

SUPPLEMENTARY TABLES

Supplementary Table 1. ASHS-T1 structural measurements, separated by group.

	**PRC thickness (mm)**		**ERC thickness (mm)**		**PHC thickness (mm)**		**Anterior HP volume (cm^3)**		**Posterior HP volume (cm^3)**	
	Left (SD)	Right (SD)	Left (SD)	Right (SD)	Left (SD)	Right (SD)	Left (SD)	Right (SD)	Left (SD)	Right (SD)
Young and Middle-Aged	2.37 (0.22)	2.36 (2.34)	1.97 (0.18)	2.02 (0.15)	2.13 (0.16)	2.18 (0.21)	1.73 (0.26)	1.86 (0.28)	1.83 (0.20)	1.79 (0.20)
Aβ− CU	2.31 (0.18)	2.29 (0.21)	2.05 (0.18)	2.08 (0.20)	2.04 (0.14)	2.08 (0.16)	1.64 (0.25)	1.74 (0.24)	1.68 (0.19)	1.66 (0.18)
Aβ+ CU	2.27 (0.20)	2.33 (0.13)	2.06 (0.16)	2.09 (0.14)	1.98 (0.22)	2.08 (0.19)	1.66 (0.28)	1.79 (0.28)	1.61 (0.18)	1.57 (0.20)
Aβ+ CI	2.10 (0.23)	2.17 (0.22)	1.83 (0.26)	1.87 (0.22)	1.95 (0.20)	1.95 (0.20)	1.39 (0.26)	1.47 (0.26)	1.34 (0.23)	1.37 (0.22)

Supplementary Table 2. MTL voxelwise temporal Signal-to-Noise Ratio (tSNR) for raw and preprocessed fMRI datasets, separated by

	PRC		ERC		PHC		Anterior HP		Posterior HP		$aMTL_{tau}$		$pMTL_{tau}$	
	Left (SD)	Right (SD)	Left (SD)	Right (SD)	Left (SD)	Right (SD)	Left (SD)	Right (SD)	Left (SD)	Right (SD)	Left (SD)	Right (SD)	Left (SD)	Right (SD)
Raw fMRI Data														
Young and Middle-Aged	13.63 (2.62)	13.44 (2.34)	9.55 (2.20)	10.15 (2.16)	21.90 (2.57)	21.62 (2.31)	16.97 (3.27)	18.10 (3.18)	21.74 (2.33)	21.82 (2.20)	14.27 (2.03)	15.49 (2.06)	20.83 (2.74)	20.60 (2.50)
Aβ− CU	12.48 (2.49)	11.91 (2.42)	8.92 (2.08)	9.29 (2.57)	19.45 (3.13)	18.86 (3.09)	16.14 (2.65)	16.81 (2.77)	20.61 (2.22)	20.35 (2.34)	13.55 (2.09)	14.41 (2.28)	19.52 (2.55)	19.12 (2.60)
Aβ+ CU	12.70 (2.08)	12.29 (2.34)	7.92 (1.86)	8.94 (2.44)	19.78 (2.38)	19.73 (2.52)	15.69 (3.48)	16.73 (3.61)	20.77 (2.19)	20.90 (2.18)	13.14 (1.91)	14.08 (2.51)	19.83 (2.25)	19.70 (2.01)
Aβ+ CI	12.00 (2.10)	11.56 (2.03)	7.95 (1.54)	8.90 (2.21)	19.13 (2.08)	18.61 (2.07)	15.33 (2.57)	15.99 (3.12)	20.47 (1.90)	20.36 (1.96)	13.63 (1.69)	14.43 (2.07)	19.44 (2.10)	19.33 (2.04)
Preprocessed fMRI Data														
Young and Middle-Aged	263.3 (51.5)	260.5 (51.3)	194.9 (40.1)	202.4 (34.5)	351.4 (47.3)	344.0 (45.7)	303.5 (58.6)	317.4 (55.2)	368.6 (41.4)	373.2 (39.8)	262.9 (46.8)	280.2 (44.9)	364.2 (51.0)	364.5 (44.1)
Aβ− CU	247.1 (47.3)	236.6 (48.9)	192.5 (32.4)	198.7 (35.8)	326.7 (38.9)	319.6 (46.8)	300.0 (42.2)	309.0 (42.6)	362.2 (39.2)	357.2 (38.8)	257.4 (38.0)	268.8 (38.9)	354.2 (45.5)	347.3 (47.0)
Aβ+ CU	244.8 (44.3)	240.0 (51.4)	176.9 (33.0)	190.5 (39.6)	322.1 (33.4)	321.5 (30.8)	296.8 (48.1)	309.7 (53.2)	362.3 (40.6)	365.5 (32.7)	253.4 (39.6)	264.8 (49.2)	354.8 (40.4)	352.7 (34.6)
Aβ+ CI	243.0 (43.4)	232.8 (42.4)	193.8 (28.1)	198.4 (40.0)	337.2 (42.4)	326.9 (35.4)	317.7 (47.7)	320.9 (52.3)	381.2 (46.9)	384.7 (40.7)	272.9 (38.0)	280.2 (41.9)	374.8 (51.7)	373.3 (46.6)

Supplementary Table 3. Framewise displacement (FD) for raw and filtered realignment parameters, separated by group.

	**Young and Middle-Aged (SD)**	**Aβ− CU (SD)**	**Aβ+ CU (SD)**	**Aβ+ CI (SD)**
Raw mean FD (mm)	0.1588 (0.0700)	0.2627 (0.1236)	0.2303 (0.0814)	0.2158 (0.0974)
Raw max FD (mm)	0.6209 (0.5888)	1.1046 (1.0069)	1.0697 (0.7293)	1.2868 (1.0637)
Filtered mean FD (mm)	0.0213 (0.0118)	0.0403 (0.0193)	0.0420 (0.0173)	0.0401 (0.0263)
Filtered max FD (mm)	0.1698 (0.1354)	0.2273 (0.1900)	0.2633 (0.1737)	0.2915 (0.2155)

SUPPLEMENTARY REFERENCES

Asman, A. J., & Landman, B. A. (2013). Non-local statistical label fusion for multi-atlas segmentation. *Medical Image Analysis*, *17*(2), 194-208. https://doi.org/https://doi.org/10.1016/j.media.2012.10.002

Avants, B., & Gee, J. C. (2004). Geodesic estimation for large deformation anatomical shape averaging and interpolation. *Neuroimage*, *23 Suppl 1*, S139-150. https://doi.org/10.1016/j.neuroimage.2004.07.010

Avants, B. B., Epstein, C. L., Grossman, M., & Gee, J. C. (2008). Symmetric diffeomorphic image registration with cross-correlation: evaluating automated labeling of elderly and neurodegenerative brain. *Med Image Anal*, *12*(1), 26-41. https://doi.org/10.1016/j.media.2007.06.004

Beckmann, C. F., & Smith, S. M. (2004). Probabilistic independent component analysis for functional magnetic resonance imaging. *IEEE Trans Med Imaging*, *23*(2), 137-152. https://doi.org/10.1109/TMI.2003.822821

Benjamini, Y., & Hochberg, Y. (1995). Controlling the False Discovery Rate: A Practical and Powerful Approach to Multiple Testing. *Journal of the Royal Statistical Society. Series B (Methodological)*, *57*(1), 289-300. http://www.jstor.org.proxy.library.upenn.edu/stable/2346101

Dale, A. M., Fischl, B., & Sereno, M. I. (1999). Cortical surface-based analysis. I. Segmentation and surface reconstruction. *Neuroimage*, *9*(2), 179-194. https://doi.org/10.1006/nimg.1998.0395

Fan, J., Feng, Y., & Wu, Y. (2009). Network Exploration Via the Adaptive Lasso and Scad Penalties. *Ann Appl Stat*, *3*(2), 521-541. https://doi.org/10.1214/08-AOAS215SUPP

Fan, J., & Li, R. (2001). Variable Selection via Nonconcave Penalized Likelihood and its Oracle Properties. *Journal of the American Statistical Association*, *96*(456), 1348-1360. https://doi.org/10.1198/016214501753382273

Gorgolewski, K., Burns, C. D., Madison, C., Clark, D., Halchenko, Y. O., Waskom, M. L., & Ghosh, S. S. (2011). Nipype: a flexible, lightweight and extensible neuroimaging data processing framework in python. *Front Neuroinform*, *5*, 13. https://doi.org/10.3389/fninf.2011.00013

Griffanti, L., Douaud, G., Bijsterbosch, J., Evangelisti, S., Alfaro-Almagro, F., Glasser, M. F., Duff, E. P., Fitzgibbon, S., Westphal, R., Carone, D., Beckmann, C. F., & Smith, S. M. (2017). Hand classification of fMRI ICA noise components. *Neuroimage*, *154*, 188-205. https://doi.org/10.1016/j.neuroimage.2016.12.036

Griffanti, L., Salimi-Khorshidi, G., Beckmann, C. F., Auerbach, E. J., Douaud, G., Sexton, C. E., Zsoldos, E., Ebmeier, K. P., Filippini, N., Mackay, C. E., Moeller, S., Xu, J., Yacoub, E., Baselli, G., Ugurbil, K., Miller, K. L., & Smith, S. M. (2014). ICA-based artefact removal and accelerated fMRI acquisition for improved resting state network imaging. *Neuroimage*, *95*, 232-247. https://doi.org/10.1016/j.neuroimage.2014.03.034

Hrybouski, S., Cribben, I., McGonigle, J., Olsen, F., Carter, R., Seres, P., Madan, C. R., & Malykhin, N. V. (2021). Investigating the effects of healthy cognitive aging on brain functional connectivity using 4.7 T resting-state functional magnetic resonance imaging. *Brain Struct Funct*, *226*(4), 1067-1098. https://doi.org/10.1007/s00429-021-02226-7

Hsieh, C.-J., Sustik, M. A., Dhillon, I. S., & Ravikumar, P. (2014). QUIC: quadratic approximation for sparse inverse covariance estimation. *J. Mach. Learn. Res.*, *15*, 2911-2947.

Jenkinson, M., Bannister, P., Brady, M., & Smith, S. (2002). Improved optimization for the robust and accurate linear registration and motion correction of brain images. *Neuroimage*, *17*(2), 825-841. https://doi.org/10.1016/s1053-8119(02)91132-8

Landau, S. M., Breault, C., Joshi, A. D., Pontecorvo, M., Mathis, C. A., Jagust, W. J., Mintun, M. A., & Alzheimer's Disease Neuroimaging, I. (2013). Amyloid-beta imaging with Pittsburgh compound B and florbetapir: comparing radiotracers and quantification methods. *J Nucl Med*, *54*(1), 70-77. https://doi.org/10.2967/jnumed.112.109009

Pruim, R. H. R., Mennes, M., van Rooij, D., Llera, A., Buitelaar, J. K., & Beckmann, C. F. (2015). ICA-AROMA: A robust ICA-based strategy for removing motion artifacts from fMRI data. *Neuroimage*, *112*, 267-277. https://doi.org/10.1016/j.neuroimage.2015.02.064

Schaefer, A., Kong, R., Gordon, E. M., Laumann, T. O., Zuo, X. N., Holmes, A. J., Eickhoff, S. B., & Yeo, B. T. T. (2018). Local-Global Parcellation of the Human Cerebral Cortex from Intrinsic Functional Connectivity MRI. *Cereb Cortex*, *28*(9), 3095-3114. https://doi.org/10.1093/cercor/bhx179

Smith, S. M., Jenkinson, M., Woolrich, M. W., Beckmann, C. F., Behrens, T. E., Johansen-Berg, H., Bannister, P. R., De Luca, M., Drobnjak, I., Flitney, D. E., Niazy, R. K., Saunders, J., Vickers, J., Zhang, Y., De Stefano, N., Brady, J. M., & Matthews, P. M. (2004). Advances in functional and structural MR image analysis and implementation as FSL. *Neuroimage*, *23 Suppl 1*, S208-219. https://doi.org/10.1016/j.neuroimage.2004.07.051

Smith, S. M., & Nichols, T. E. (2009). Threshold-free cluster enhancement: addressing problems of smoothing, threshold dependence and localisation in cluster inference. *Neuroimage*, *44*(1), 83-98. https://doi.org/10.1016/j.neuroimage.2008.03.061

Tustison, N. J., Avants, B. B., Cook, P. A., Zheng, Y., Egan, A., Yushkevich, P. A., & Gee, J. C. (2010). N4ITK: improved N3 bias correction. *IEEE Trans Med Imaging*, *29*(6), 1310-1320. https://doi.org/10.1109/TMI.2010.2046908

Wang, H., Suh, J. W., Das, S. R., Pluta, J. B., Craige, C., & Yushkevich, P. A. (2013). Multi-Atlas Segmentation with Joint Label Fusion. *IEEE Trans Pattern Anal Mach Intell*, *35*(3), 611-623. https://doi.org/10.1109/TPAMI.2012.143

Whitfield-Gabrieli, S., & Nieto-Castanon, A. (2012). Conn: a functional connectivity toolbox for correlated and anticorrelated brain networks. *Brain Connect*, *2*(3), 125-141. https://doi.org/10.1089/brain.2012.0073

Zhang, Y., Brady, M., & Smith, S. (2001). Segmentation of brain MR images through a hidden Markov random field model and the expectation-maximization algorithm. *IEEE Trans Med Imaging*, *20*(1), 45-57. https://doi.org/10.1109/42.906424

Zhu, Y., & Cribben, I. (2018). Sparse Graphical Models for Functional Connectivity Networks: Best Methods and the Autocorrelation Issue. *Brain Connect*, *8*(3), 139-165. https://doi.org/10.1089/brain.2017.0511
